# Relationship Between Plasma Homocysteine and Bone Density, Lean Mass, Muscle Strength and Physical Function in 1480 Middle-Aged and Older Adults: Data from NHANES

**DOI:** 10.1007/s00223-022-01037-0

**Published:** 2022-11-07

**Authors:** Jatupol Kositsawat, Sara Vogrin, Chloe French, Maria Gebauer, Darren G. Candow, Gustavo Duque, Ben Kirk

**Affiliations:** 1 Center on Aging, University of Connecticut, Farmington, CT USA; 2 Australian Institute for Musculoskeletal Science (AIMSS), The University of Melbourne and Western Health, 176 Furlong Road, St. Albans, VIC 3021 Australia; 3 Department of Medicine-Western Health, Melbourne Medical School, The University of Melbourne, St Albans, VIC 3021 Australia; 4 School of Health Sciences, University of Manchester, Manchester, UK; 5 University Hospital of Guadalajara, Guadalajara, Spain; 6 Faculty of Kinesiology and Health Studies, University of Regina, Regina, Canada

**Keywords:** Homocysteine, Bone fragility, Muscle weakness, Catabolism, Sarcopenia

## Abstract

Hyperhomocysteinemia induces oxidative stress and chronic inflammation (both of which are catabolic to bone and muscle); thus, we examined the association between homocysteine and body composition and physical function in middle-aged and older adults. Data from the National Health and Nutrition Examination Survey was used to build regression models. Plasma homocysteine (fluorescence immunoassay) was used as the exposure and bone mineral density (BMD; dual-energy X-ray absorptiometry; DXA), lean mass (DXA), knee extensor strength (isokinetic dynamometer; newtons) and gait speed (m/s) were used as outcomes. Regression models were adjusted for confounders (age, sex, race/Hispanic origin, height, fat mass %, physical activity, smoking status, alcohol intakes, cardiovascular disease, diabetes, cancer and vitamin B12). All models accounted for complex survey design by using sampling weights provided by NHANES. 1480 adults (median age: 64 years [IQR: 56, 73]; 50.3% men) were included. In multivariable models, homocysteine was inversely associated with knee extensor strength (*β* = 0.98, 95% CI 0.96, 0.99, *p* = 0.012) and gait speed (*β* = 0.85, 95% CI 0.78, 0.94, *p* = 0.003) and borderline inversely associated with femur BMD (*β* = 0.84, 95% CI 0.69, 1.03, *p* = 0.086). In the sub-group analysis of older adults (≥ 65 years), homocysteine was inversely associated with gait speed and femur BMD (*p* < 0.05) and the slope for knee extensor strength and whole-body BMD were in the same direction. No significant associations were observed between homocysteine and total or appendicular lean mass in the full or sub-group analysis. We found inverse associations between plasma homocysteine and muscle strength/physical function, and borderline significant inverse associations for femur BMD.

## Introduction

Homocysteine, a non-essential amino acid biosynthesised from methionine during demethylation, is an important signalling molecule for gene expression of proteins and enzymes in the human body [1]. When maintained in normal ranges, homocysteine serves as an important cofactor in metabolic processes; however, abnormally high plasma/serum levels (termed hyperhomocysteinemia) are toxic to the body. More specifically, chronic hyperhomocysteinemia damages cells and tissues and induces oxidative stress and chronic inflammation in the body [2, 3], as well as disrupting the vasodilatory properties of nitric oxide in various tissues [4].

As a result, hyperhomocysteinemia has been identified as a risk factor for several age-related pathologies including cardiovascular diseases, diabetes and dementia [5–7]. Causes of elevated homocysteine levels include; but are not limited to, old age, sex (primarily men), smoking status, excess alcohol intakes, deficiencies in enzymes (i.e. Cystathionin/Methionine-β-synthase) and vitamins (folate, vitamin B6 and B12), genetic polymorphisms, and several pathologies (i.e. cancers, renal dysfunction, systemic lupus erythematosus) [8–10].

Given that hyperhomocysteinemia induces oxidative stress and chronic inflammation (both of which are catabolic to bone and muscle [11, 12]), it is not surprising that epidemiological studies have focussed on the link between homocysteine and musculoskeletal health. Some studies report positive relationships between homocysteine levels and the loss of muscle mass and function (sarcopenia) [13, 14] while others show no relationship [15]. These ambiguous findings are further supported by a 2021 narrative review which concluded that results between homocysteine levels and muscle function are inconclusive and warrant further research [16]. Mechanistically, hyperhomocysteinemia is suggested to adversely impact the structure and function of skeletal muscle by inducing oxidative stress which in turn leads to mitochondrial loss [2], or by decreasing the bioavailability of nitric oxide and blood flow to muscle cells [17].

Regarding low to very-low bone density (osteopenia/osteoporosis), findings are equally heterogeneous. A meta-analysis conducted in 2014 found that homocysteine levels were significantly higher in postmenopausal women with osteoporosis [18]; however, a later meta-analysis (2021) [19] found no such association. A recent (2022) cross-sectional study [20] examined the relationship between homocysteine and bone density (at femoral neck, spine and hip) in 760 postmenopausal women and also found no association. The suggested biological link between homocysteine and bone fragility stems from the role of this molecule in modulating osteoclastogenesis (bone breakdown) and decreasing blood flow in this tissue through its actions on nitric oxide [21].

Further research is still needed to examine the relationship between homocysteine and musculoskeletal health using large cohort studies among adults of different race/Hispanic origin. These studies should also include multiple measures of musculoskeletal health including bone density and muscle mass and function which are inherently linked [22]. Given this, we sought to build on current work and re-examine the relationship between plasma homocysteine and bone density, lean mass, muscle strength and physical function using data from middle-aged and older adults in the National Health and Nutrition Examination Survey (NHANES).

## Materials and Methods

### Population

We studied individuals participating in the 2001–2002 NHANES as it contained the outcomes of interest. The NHANES is a population-representative sample aimed at assessing the health and nutritional status of non-institutionalized civilians residing in the United States. From the 2001 to 2002 NHANES database, there were 11,039 participants in total. Plasma homocysteine data was missing in 2567 participants and DXA was not performed in 321 pregnant women. A further 966 participants had missing DXA data, leaving 7185 participants that had DXA scans. Exclusion criteria for DXA scan in NHANES included; pregnant women, or participants with a height above 196 cm (DXA table length) or weight above 136 kg (DXA table weight limit) [23]. Of note, even though DXA scan was performed in all ages, muscle strength and physical function were performed only in participants ≥ 50 years. In addition, participants with recent myocardial infarction (past 6 weeks), brain aneurysm/stroke, chest/abdominal surgery (past 3 weeks), knee surgery or severe back pain were excluded from strength testing. Since our research aimed at the associations on both muscle and bone health, out of 7185 participants, we included only *n* = 1480 participants that had full DXA scan (lean mass, bone density, fat mass) along with data on muscle strength and physical function in the final analytical sample. Given that homocysteine levels are higher and musculoskeletal health is deteriorating with age, we also performed sub-group analyses of adults aged ≥ 65 years comprising of *n* = 735 older adults. All participants provided written informed consent in accordance with the Centers for Disease Control and Prevention (CDC). Ethical approval for NHANES (1999–2004) was received from the NCHS Research Ethics Review Board (ERB): Protocol #98–12.

### Plasma Homocysteine

As previously described [24], total plasma homocysteine was measured on the Abbott Homocysteine IMX in 2001 and Abbott Axsym in 2002, both a fully automated fluorescence polarization immunoassay. This method has shown to have excellent precision (coefficient of variation: ≤ 5%) when compared to high-performance liquid chromatography [25]. Age- and sex-specific reference ranges for homocysteine were reported following the 2001–2002 NHANES CDC data [24].

### Anthropometry and Body Composition

Height (cm.) and weight (kg.) were measured using standardized procedures [26]. Whole-body DXA scans were performed on a QDR 4500A fan beam densitometer (Hologic, Inc., Bedford, MA) following manufactures guidelines. We reported bone mineral density (BMD) in g/cm^2^. Participants changed into gowns and removed any jewelry or metal objects which could interfere with the scan result. We used available data for total and regional bone mineral density (BMD) and lean mass (the sum of non-bone and non-fat masses in kg and %). Of note, the NHANES DXA lean mass and fat mass were adjusted based on the results of an analysis of QDR-4500A DXA data. The lean mass was decreased by 5% and an equivalent kg weight was added to the fat mass.

### Muscle Strength and Physical Function

#### Muscle Strength

Average peak force of the knee extensors was measured using a Kin Com Isokinetic Dynamometer (Chattanooga group, Inc, Chattanooga, TN). Participants completed six repetitions (3 familiarization, 3 testing) with the highest value (in Newtons) from the final 3 tests used in the analysis. Where less than 4 trials were completed, the highest value from the remaining attempts was used in the analysis.

#### Physical Function

Participants completed a timed 20 feet (6.1 m) walk at usual speed. If needed, a cane or walker was permitted. Gait speed was then transformed into meters per second (m/s) and used in the analysis. The full protocol for these procedures is available elsewhere [27].

### Demographics, Lifestyle, and Medical Conditions

Demographic, lifestyle, and medical conditions were recorded via self-reported questionnaires. We included data on demographics (age, sex, race/Hispanic origin), history of chronic illness (including diabetes, congestive heart failure, coronary heart disease, stroke, and cancer), physical activity levels, and lifestyle factors including smoking status and alcohol intakes. These methods have been described in previous NHANES articles [28, 29].

### Statistical Methods

Statistical analyses were performed using Stata 16.1 (StataCorp. 2019. Stata Statistical Software: Release 16. College Station, TX: StataCorp LLC.). Data were presented as frequency (percentage [%]) for categorical variables or median (interquartile range [IQR]) for continuous variables. Scatter plots were used to visualize the relationship between the exposure and outcomes of interest. Outcomes of interest were: total BMD (in g/cm^2^ and T score), lumbar spine and femur BMD (both in g/cm^2^), total and appendicular lean mass (both in kg), knee extensor strength (in Newtons) and gait speed (in m/s). In these data, fit of linear regression (assessed by visual inspection of residuals) was better when homocysteine was log transformed. As such, exponentiated beta ($\beta$) coefficients with 95% CI represent fold difference in homocysteine as the variable increases for 1 unit. Univariable linear regression was first used to assess associations between homocysteine and other variables. Regardless of univariable results, all multivariable models were adjusted for all pre-specified confounders, which were chosen based on current literature. These are: age, sex, race/Hispanic origin, smoking status, alcohol intakes, height, DXA fat mass (%), physical activity, cardiovascular disease, diabetes, cancer and vitamin B12. Missing DXA data were imputed using sequential regression imputation method (further description available in NHANES documentation) [30]. Coefficients and standard errors for all analyses were adjusted for the variability between imputations according to the combination rules by Rubin [31]. All regression results also took into account complex survey design by using sampling weights (medical examination clinic weights [32] provided by NHANES) and Taylor linearized variance estimation. All analyses were repeated in a sub-group of older adults (≥ 65 years old). A *p*-value < 0.05 was considered statistically significant and a *p*-value between 0.05 and 0.10 was considered borderline significant.

## Results

### Study Population

Median age of participants was 64 years (IQR: 56, 73) with a relatively equal proportion of men (50.3%) and women (49.7%) in the sample. Median plasma concentration of homocysteine was 9.15 µmol/L (IQR: 7.58, 11.24). According to age- and sex-specific reference ranges for homocysteine, over two-thirds (70.9%) of participants had normal levels, 26.3% had mild to severe hyperhomocysteinemia, and 2.8% had hypohomocysteinemia (Table 1). Tables 1 and 2 showed the participants characteristics in the full population (≥ 50 years) and the sub-group of older adults (≥ 65 years), respectively. Compared to the full population, the prevalence of moderate to severe hyperhomocysteinemia tended to be higher in older adults (8.7% vs 5.5%, *p* < 0.001; Tables 1 and 2). Compared to the full population, older adults have significantly lower BMD, appendicular lean mass and knee extensor strength as well as slower gait speed (*p* < 0.001 for all).

**Table 1:** Participant characteristics and univariable associations between homocysteine and potential confounders (demographic, lifestyle and clinical factors) in middle-aged and older adults (≥ 50 years) (*n* = 1480)

Variable	Median [IQR] or *n* (%)	$\beta$[95% CI]	*p* value
Age (years)	64 (56, 73)	1.01 [1.01, 1.01]	< 0.001
Sex			
Men	745 (50.3%)	Reference	
Women	735 (49.7%)	0.88 [0.86, 0.90]	< 0.001
Homocysteine (µmol/L)	9.15 (7.58, 11.24)		
Below range	41 (2.8%)		
Normal range	1050 (70.9%)		
Mild hyperhomocysteinemia	307 (20.7%)		
Moderate hyperhomocysteinemia	73 (4.9%)		
Intermediate hyperhomocysteinemia	8 (0.5%)		
Severe hyperhomocysteinemia	1 (0.1%)		
Race/Hispanic origin			
Mexican American	222 (15.0%)	Reference	
Other Hispanic	43 (2.9%)	1.09 [0.95, 1.24]	0.193
Non-Hispanic White	943 (63.7%)	1.04 [0.96, 1.13]	0.279
Non-Hispanic Black	233 (15.7%)	1.09 [0.99, 1.19]	0.069
Other race	39 (2.6%)	0.97 [0.87, 1.09]	0.569
Weight (kg)	77.2 (66.1, 89.6)	1 [1.00, 1.00]	0.183
Height (m)	1.67 (1.60, 1.75)	1 [1.00, 1.01]	0.001
Body Mass Index (kg/m^2^)	27.5 (24.5, 30.9)	1 [1.00, 1.01]	0.889
Moderate activity over past 30 days			
No	776 (52.4%)	Reference	
Yes	664 (44.9%)	0.92 [0.89, 0.96]	0.001
Unable to do	39 (2.6%)	1.18 [0.98, 1.42]	0.079
Unknown	1 (0.1%)	0.82 [0.80, 0.85]	< 0.001
Smoking status			
Non-smoker	575 (38.9%)	Reference	
Current smoker	226 (15.3%)	1.07 [1.01, 1.13]	0.03
Unknown	679 (45.9%)	0.99 [0.95, 1.02]	0.431
Number of drinking days over last 12 months			
Never	365 (24.7%)	Reference	
1–2 times	400 (27.0%)	0.99 [0.93, 1.05]	0.732
3–10 times	410 (27.7%)	1.03 [0.96, 1.09]	0.373
> 10 times	55 (3.7%)	1.09 [1.01, 1.18]	0.024
Unknown	250 (16.9%)	1.04 [0.95, 1.14]	0.38
Ever told you had congestive heart failure			
No	1419 (95.9%)	Reference	
Yes	51 (3.4%)	1.26 [1.13, 1.40]	0.001
Unknown	10 (0.7%)	1.25 [0.99, 1.58]	0.063
Ever told you had coronary heart disease			
No	1354 (91.5%)	Reference	
Yes	114 (7.7%)	1.18 [1.10, 1.26]	< 0.001
Unknown	12 (0.8%)	1.14 [0.95, 1.38]	0.14
Ever told you had a stroke			
No	1461 (98.7%)	Reference	
Yes	15 (1.0%)	1.20 [0.97, 1.49]	0.089
Unknown	4 (0.3%)	1.18 [0.89, 1.55]	0.227
Doctor told you have diabetes			
No	1242 (83.9%)	Reference	
Yes	238 (16.1%)	1.08 [1.03, 1.13]	0.003
Ever told you had cancer			
No	1250 (84.5%)	Reference	
Yes	229 (15.5%)	1 [0.97, 1.04]	0.835
Unknown	1 (0.1%)	0.67 [0.65, 0.68]	< 0.001
Total fat mass (kg)*	26.68 (21.41, 33.36)	0.99 [0.97, 1.01]	0.442
Total fat mass (%)^+^	35.1 (29.2, 41.7)	0.96 [0.95, 0.98]	< 0.001
Vitamin B12, serum (pg/mL)	478 (358, 633)	1.00 [1.00, 1.00]	0.009

Coefficient ($\beta$) with 95% CI represents fold difference in homocysteine as the variable increases for 1 unit. *Increase for 10 kg. ^+^Increase for 10%

**Table 2:** Participant characteristics and univariable associations between homocysteine and potential confounders (demographic, lifestyle and clinical factors) in older adults (≥ 65 years) (*n* = 735)

Variable	Median [IQR] or *n* (%)	$\beta$[95% CI]	*p* value
Age (years)	73 (69, 80)	1.01 [1.01, 1.02]	< 0.001
Sex			
Men	368 (50.1%)	Reference	
Women	367 (49.9%)	0.9 [0.85, 0.95]	0.002
Homocysteine (µmol/L)	9.84 (8.15, 12.15)		
Below range	16 (2.2%)		
Normal range	522 (71.0%)		
Mild hyperhomocysteinemia	133 (18.1%)		
Moderate hyperhomocysteinemia	57 (7.8%)		
Intermediate hyperhomocysteinemia	6 (0.8%)		
Severe hyperhomocysteinemia	1 (0.1%)		
Race/Hispanic origin			
Mexican American	102 (13.9%)	Reference	
Other Hispanic	19 (2.6%)	1.18 [1.01, 1.39]	0.043
Non-Hispanic White	497 (67.6%)	1.13 [1.02, 1.26]	0.028
Non-Hispanic Black	107 (14.6%)	1.15 [1.00, 1.31]	0.048
Other race	10 (1.4%)	1.12 [0.95, 1.33]	0.161
Weight (kg)	73.7 (63.9, 84.4)	1 [1.00, 1.00]	0.249
Height (m)	1.65 (1.58, 1.73)	1 [1.00, 1.01]	0.135
Body Mass Index (kg/m^2^)	26.9 (24.0, 29.8)	1 [0.99, 1.01]	0.591
Moderate activity over past 30 days			
No	376 (51.2%)	Reference	
Yes	329 (44.8%)	0.89 [0.84, 0.95]	0.001
Unable to do	29 (3.9%)	1.15 [0.84, 1.57]	0.349
Unknown	1 (0.1%)	0.72 [0.69, 0.76]	< 0.001
Smoking status			
Non-smoker	300 (40.8%)	Reference	
Current smoker	69 (9.4%)	1.01 [0.88, 1.17]	0.874
Unknown	366 (49.8%)	0.95 [0.91, 1.00]	0.054
Number of drinking days over last 12 months			
Never	187 (25.4%)	Reference	
1–2 times	173 (23.5%)	1.01 [0.92, 1.12]	0.785
3–10 times	196 (26.7%)	1.01 [0.92, 1.11]	0.801
> 10 times	25 (3.4%)	0.99 [0.84, 1.17]	0.88
Unknown	154 (21.0%)	1.07 [0.97, 1.18]	0.164
Ever told you had congestive heart failure			
No	691 (94.0%)	Reference	
Yes	35 (4.8%)	1.26 [1.16, 1.36]	< 0.001
Unknown	9 (1.2%)	1.14 [0.88, 1.49]	0.295
Ever told you had coronary heart disease			
No	650 (88.4%)	Reference	
Yes	75 (10.2%)	1.21 [1.11, 1.31]	< 0.001
Unknown	10 (1.4%)	1.05 [0.82, 1.34]	0.706
Ever told you had a stroke			
No	720 (98.0%)	Reference	
Yes	11 (1.5%)	1.24 [0.96, 1.60]	0.099
Unknown	4 (0.5%)	1.05 [0.80, 1.39]	0.687
Doctor told you have diabetes			
No	605 (82.3%)	Reference	
Yes	130 (17.7%)	1.09 [0.99, 1.19]	0.072
Ever told you had cancer			
No	582 (79.2%)	Reference	
Yes	153 (20.8%)	0.98 [0.91, 1.05]	0.496
Total fat mass (kg)*	25.9 (20.8, 32.2)	1 [0.97, 1.04]	0.788
Total fat mass (%)^+^	35.4 (30.0, 41.5)	0.98 [0.95, 1.01]	0.197
Vitamin B12, serum (pg/mL)	478 (353, 639)	1.00 [1.00, 1.00]	< 0.001

Coefficient ($\beta$) with 95% CI represents fold difference in homocysteine as the variable increases for 1 unit. *Increase for 10 kg. ^+^Increase for 10%

### Univariable Associations Between Homocysteine and Demographic, Lifestyle, and Medical Conditions

In univariable analyses of the full population (Table 1), homocysteine was associated with age, sex, height, smoking status, alcohol intakes, physical activity and total fat mass (%), as well as certain diseases including coronary heart disease, congestive heart failure, and diabetes.

### Univariable- and Multivariable-Associations Between Homocysteine and Outcome Measures

#### Muscle Outcome Measures

**Fig. 1:**
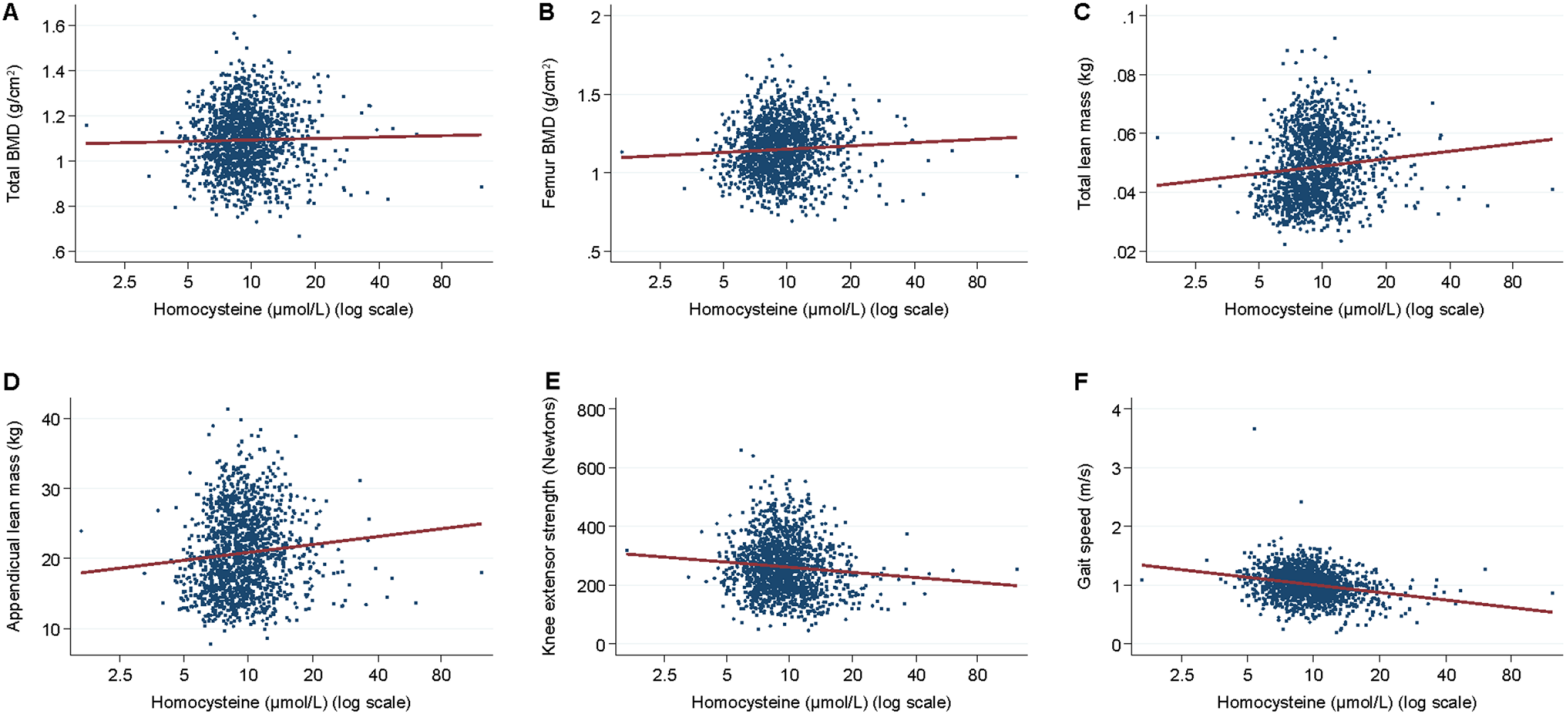
Scatter plots shows univariable (unadjusted) associations between homocysteine and outcome measures (*n* = 1480)

In univariable analyses of the full population, homocysteine was positively associated with total lean mass and appendicular lean mass and inversely associated with knee extensor strength and gait speed (*p* < 0.05; Fig. 1 and Table 3). In the fully adjusted model, homocysteine was inversely associated with knee extensor strength (*β* = 0.98, 95% CI 0.96, 0.99, *p* = 0.012) and gait speed (*β* = 0.85, 95% CI 0.78, 0.94, *p* = 0.003). No significant associations (*p* ≥ 0.290 to 0.316) were observed between homocysteine and total/appendicular lean mass in the full population.

**Table 3:** Univariable- and multivariable- associations between homocysteine and outcome measures (*n* = 1480)

Variable	Median (IQR)	Model 1		Model 2		Model 3	
		$\beta$[95% CI]	*P* value	$\beta$[95% CI]	*P* value	$\beta$[95% CI]	*P* value
Total BMD (g/cm^2^)	1.09 (1.00, 1.18)	0.93 [0.76, 1.14]	0.469	0.89 [0.71, 1.11]	0.266	0.90 [0.70, 1.15]	0.361
Total BMD, *T* score	0.68 (− 0.09, 1.39)	0.99 [0.97, 1.02]	0.469	0.98 [0.96, 1.01]	0.266	0.99 [0.96, 1.02]	0.361
Lumbar-Spine BMD (g/cm^2^)	0.99 (0.88, 1.12)	1.03 [0.88, 1.20]	0.684	0.99 [0.88, 1.12]	0.841	0.96 [0.85, 1.08]	0.448
Femur BMD (g/cm^2^)	1.15 (1.03, 1.26)	1.03 [0.89, 1.19]	0.643	0.85 [0.71, 1.02]	0.08	**0.84 [0.69, 1.03]**	**0.086**
Total lean mass (kg)*	47.4 (39.2, 57.5)	1.03 [1.01, 1.05]	0.009	1.01 [0.98, 1.04]	0.49	0.98 [0.94, 1.02]	0.290
Appendicular lean mass (kg)*	20.3 (16.1, 25.0)	1.01 [1.00, 1.01]	0.009	1.00 [1.00, 1.01]	0.475	1.00 [0.99, 1.00]	0.316
Knee extensor strength (N)^+^	253 (196, 318)	0.97 [0.95, 1.00]	0.034	0.97 [0.95, 0.99]	0.011	**0.98 [0.96, 0.99]**	**0.012**
Gait speed (m/s)	1.02 (0.86, 1.18)	0.71 [0.65, 0.78]	< 0.001	0.80 [0.73, 0.88]	< 0.001	**0.85 [0.78, 0.94]**	**0.003**

*BMD* Bone mineral density. Coefficient ($\beta$) with 95% CI represents fold difference in homocysteine as the variable increases for 1 unit. *Increase for 10 kg. ^+^Increase for 100 N. Model 1: unadjusted; Model 2: adjusted for age and sex. Model 3: age, sex, race/Hispanic origin, smoking status, alcohol intakes, height, fat mass (%), physical activity, cardiovascular disease, diabetes, cancer and vitamine B12. All regressions were also accounted for complex survey design using sampling weights provided. Bolded values statistically significant or borderline significant in Model 3

In the sub-group analysis of older adults, the inverse associations between homocysteine and gait speed remained (*β* = 0.80, 95% CI 0.70, 0.91, *p* = 0.003) in the fully adjusted model. Associations for knee extensor strength were attenuated to non-significance (*β* = 0.97, 95% CI 0.94, 1.01, *p* = 0.112) in older adults, although the slope was in the same direction.

#### Bone Outcome Measures

In the fully adjusted model, homocysteine was borderline inversely associated with femur BMD (*β* = 0.84, 95% CI: 0.69, 1.03, *p* = 0.086). No significant associations (*p* ≥ 0.361 to 0.448) were observed between homocysteine and total/lumbar-spine BMD in the full population.

In the sub-group of older adults, in the fully adjusted models, homocysteine was significantly, and inversely associated with femoral BMD (*β* = 0.71, 95% CI 0.56, 0.90, *p* = 0.008) and was borderline inversely associated with total body BMD (*β* = 0.78, 95% CI 0.59, 1.03, *p* = 0.080). (Table 4).

**Table 4:** Univariable- and multivariable- associations between homocysteine and outcome measures in older adults (≥ 65 years) (*n* = 735)

Variable	Median (IQR)	Model 1		Model 2		Model 3	
		$\beta$[95% CI]	*P* value	$\beta$[95% CI]	*P* value	$\beta$[95% CI]	*P* value
Total BMD (g/cm^2^)	1.05 (0.95, 1.16)	0.95 [0.76, 1.20]	0.65	0.78 [0.59, 0.99]	0.055	**0.78 [0.59, 1.03]**	**0.080**
Total BMD, *T* score	0.37 (− 0.47, 1.20)	0.99 [0.97, 1.02]	0.65	0.97 [0.94, 1.00]	0.055	**0.97 [0.94, 1.00]**	**0.080**
Lumbar-Spine BMD (g/cm^2^)	0.98 (0.85, 1.12)	1.08 [0.91, 1.28]	0.368	0.96 [0.77, 1.03]	0.525	0.90 [0.77, 1.05]	0.169
Femur BMD (g/cm^2^)	1.11 (0.98, 1.24)	1 [0.84, 1.19]	0.994	0.76 [0.56, 0.91]	0.031	**0.71 [0.56, 0.90]**	**0.008**
Total lean mass (kg)*	45.1 (37.8, 54.2)	1.03 [0.99, 1.06]	0.107	1.02 [0.91, 1.04]	0.399	0.95 [0.89, 1.02]	0.140
Appendicular lean mass (kg)	19.1 (15.3, 23.2)	1.01 [1.00, 1.01]	0.12	1.00 [0.98, 1.01]	0.388	0.99 [0.98, 1.01]	0.232
Knee extensor strength (N)^+^	224.0 (174.2, 282.5)	0.97 [0.93, 1.02]	0.193	0.97 [0.94, 1.02]	0.189	0.97 [0.94, 1.01]	0.112
Gait speed (m/s)	0.94 (0.78, 1.08)	0.7 [0.60, 0.82]	0	0.75 [0.71, 0.98]	0.003	**0.80 [0.70, 0.91]**	**0.003**

BMD: Bone mineral density. Coefficient ($\beta$) with 95% CI represents fold difference in homocysteine as the variable increases for 1 unit. *Increase for 10 kg. ^+^Increase for 100 N. Model 1: unadjusted; Model 2: adjusted for age and sex. Model 3: age, sex, race/Hispanic origin, smoking status, alcohol intakes, height, fat mass (%), physical activity, cardiovascular disease, diabetes, cancer and vitamin B12. All regressions were also accounted for complex survey design using sampling weights provided. Bolded values statistically significant or borderline significant in Model 3

## Discussion

In this population-based study of middle-aged and older adults, we found inverse associations between plasma homocysteine and muscle strength/physical function, and borderline significant inverse associations for BMD at the femur (similar patterns were observed with these outcomes in our sub-group analysis of older adults). However, we observed no association between plasma homocysteine and total or appendicular lean mass in the full population or sub-group analysis. Of note, we are the first to report on the relationship between homocysteine and bone and muscle health in the same cohort.

Various mechanisms may explain the relationship between homocysteine and muscle strength/physical function. First, hyperhomocysteinemia may increase the release of reactive oxygen species that lead to mitochondrial damage and resulting inflammation [33]. Second, hyperhomocysteinemia decreases bioavailability of nitric oxide and decreases blood flow to muscle cells [17] which may lead to lower muscle strength and physical function. Indeed, in the Baltimore Longitudinal Study of Aging, there was an inverse association between homocysteine and grip strength in healthy women $\ge$50 years over a period of 4.7 years follow up [34]. In the same study, there was inverse relationship between homocysteine and gait speed [35]. Our data strengthen these findings and support the possible role of homocysteine influencing lower-limb muscle strength and physical function, the latter of which are both crucial for healthy ageing and preventing falls and fractures [36].

Several other studies have investigated the association between homocysteine and sarcopenia definitions using muscle mass, strength and/or physical function. The Maastricht Sarcopenia Study [14] found that homocysteine levels in participants with sarcopenia (defined by the European Working Group on Sarcopenia in Older People (EWGSOP) [37]) were 27% higher compared to participants without sarcopenia. Another observational study [13] in 1582 Asian participants demonstrated that elevated homocysteine was associated with sarcopenia (defined by Asian Working Group for Sarcopenia (AWGS 2019) in community dwelling adults [38]. However, one Japanese small study [15] assessing 47 women with sarcopenia and 23 age- and sex-matched controls found no association of homocysteine levels with sarcopenia. Another European study showed that sarcopenia (defined according to EWGSOP) may be related to vitamin B12 deficiency in 403 older adults [39], even though homocysteine was not directly assessed. This is supported by another study in 66 older adults that showed that vitamin B12 was 15% lower in the sarcopenic group compared to controls [40]. A more recent study [41] conducted in type 2 diabetes mellitus patients > 60 years also found a positive correlation between homocysteine and sarcopenia (defined by the updated criteria from the EWGSOP2 [42]) independent of HbA1c levels. From these studies, it is not clear if homocysteine is linked to specific components of sarcopenia (i.e. lean mass, strength or function), but our data does not support any association between this biomarker and lean mass which includes all non-fat and non-bone body masses. Further studies should examine the association between homocysteine and direct measures of muscle mass/volume using magnetic resonance imaging, creatine dilution or high-resolution computed tomography. This will allow a better understanding of the relationship between homocysteine and muscle morphology.

In the full population, we found borderline significant and inverse associations between homocysteine and femur BMD but not whole-body or lumbar-spine BMD. In our sub-group of older adults, we found inverse associations between homocysteine and femur BMD and borderline significant inverse associations with whole-body BMD. The associations were stronger than in full population. Differences in sample sizes, bone density (which is lower in older versus younger adults), and region of interest (lumbar-spine less reliable due to stenosis/kyphosis with ageing) may account for the observed patterns. Irrespective of this, our findings add to the literature on this topic. Indeed, a meta-analysis [18] found that homocysteine levels were significantly higher in postmenopausal women with osteoporosis compared to those without osteoporosis. Another meta-analysis considering oxidative stress-related biomarkers in postmenopausal women with osteoporosis [43] showed higher levels of homocysteine in postmenopausal women with osteoporosis. However, a later meta-analysis [19] did not find higher homocysteine in postmenopausal women with osteoporosis compared to healthy controls. A recent cross-sectional study in 760 postmenopausal women with a mean age of 56 [20] also found no association between homocysteine and BMD in the lumbar spine, femoral neck and total hip. Given our findings, future research studies should re-examine this relationship using a longitudinal design to determine if any cause-and-effect exists. These studies should also include mechanisms of bone biology to confirm whether the mechanistic links were through bone formation and/or bone resorption.

A major strength of our study is the inclusion of a diverse range of men and women (and older adults) which are representative of the US population. As shown in previous studies, bone strength is different between races (BMD is higher in blacks [44]) and we found there were significant differences between the races on homocysteine levels in older adults (Table 2). The inclusion of different races in the analyses makes our results more applicable to a wider range of population. Our analysis also accounted for various demographic, lifestyle and medical factors known to impact the exposure (homocysteine) or outcome measures (bone density, lean mass, muscle strength, physical function). However, the cross-sectional nature of the study limits cause-and-effect interpretation. We also acknowledge that population-based studies are open to residual confounding. Lastly, including more accurate measures of muscle mass/size and bone structure would have strengthen our ability to examine association with homocysteine levels. Future population-based studies should consider these factors.

To conclude, in this population-based study of middle-aged and older adults, we found inverse associations between plasma homocysteine and muscle strength/physical function, and borderline inverse associations for BMD at the femur (similar patterns for these outcomes were observed in our sub-group of older adults). Longitudinal studies should now investigate the link between homocysteine and bone density, muscle strength and physical function, and these studies should consider accurate measures of muscle mass instead of lean mass.
